# Early detection of acute kidney injury in the perioperative period of liver transplant with neutrophil gelatinase-associated lipocalin

**DOI:** 10.1186/s12882-019-1566-9

**Published:** 2019-10-15

**Authors:** Camila Lima, Luciana Bertocco de Paiva Haddad, Patrícia Donado Vaz de Melo, Luiz Marcelo Malbouisson, Lilian Pires Freitas do Carmo, Luiz Augusto Carneiro D’Albuquerque, Etienne Macedo

**Affiliations:** 1 Department of Internal Medicine, Nephrology Division, University of Sao Paulo, Present Address: 419 Av. Dr Enéas de Carvalho Aguiar, third floor – room 340, 05403-000, Cerqueira Cesar, São Paulo, Brazil; 2 Department of Medical Surgical Nursing, University of Sao Paulo Nursing School, Sao Paulo, Brazil; 3 Department of Gastrointestinal Surgery, Clinical Surgery Division, University of Sao Paulo, Sao Paulo, Brazil; 4 Present Address: La Jolla, San Diego, USA; 5 Department of Biology, Biochemistry Division, Federal University of Minas Gerais, Belo Horizont, Brazil; 6 Department of Anaesthesiology, Clinical Surgery Division, University of Sao Paulo, Sao Paulo, Brazil; 7 Department of Medicine, Nephrology Division, University of California San Diego, San Diego, USA

**Keywords:** Acute kidney injury, Biomarkers, Liver transplant, Neutrophil gelatinase-associated lipocalin, Renal dialysis

## Abstract

**Background:**

Acute kidney injury (AKI) is a common complication in patients undergoing liver transplant (LT) and is associated with high morbidity and mortality. We aim to evaluate the pattern of urine and plasma neutrophil gelatinase-associated lipocalin (NGAL) elevation during the perioperative period of LT and to assess it as a prognostic marker for AKI progression, need for dialysis and mortality.

**Methods:**

We assessed NGAL levels before induction of anesthesia, after portal reperfusion and at 6, 18, 24, and 48 h after surgery. Patients were monitored daily during the first week after LT.

**Results:**

Of 100 enrolled patients undergoing liver transplant, 59 developed severe AKI based on the KDIGO serum creatinine (sCr) criterion; 34 were dialysed, and 21 died within 60 days after LT. Applying a cut-off value of 136 ng/ml, UNGAL values 6 h after surgery was a good predictor of AKI development within 7 days after surgery, having a positive predictive value (PPV) of 80% with an AUC of 0.76 (95% CI 0.67–0.86). PNGAL at 18 h after LT was also a good predictor of AKI in the first week, having a PPV of 81% and AUC of 0.74 (95% CI 0.60–0.88). Based on PNGAL and UNGAL cut-off criteria levels, time to AKI diagnosis was 28 and 23 h earlier than by sCr, respectively. The best times to assess the need for dialysis were 18 h after LT by PNGAL and 06 h after LT by UNGAL.

**Conclusion:**

In conclusion, the plasma and urine NGAL elevation pattern in the perioperative period of the liver transplant can predict AKI diagnosis earlier. UNGAL was an early independent predictor of AKI development and need for dialysis. Further studies are needed to assess whether the clinical use of biomarkers can improve patient outcomes.

**Trial registration:**

Registered at Clinical Trials (clinicaltrials.gov) in March 24th, 2014 by title “Acute Kidney Injury Biomarkers: Diagnosis and Application in Pre-operative Period of Liver Transplantation (AKIB)” and identifier NCT02095431, retrospectively registered.

## Background

Acute kidney injury (AKI) is a common complication in patients undergoing liver transplant (LT) [1]. Recent studies have shown that even mild or transient post-LT AKI is associated with prolonged intensive care or hospital stay, decreased organ survival, and increased all-cause mortality [1–3].

The etiology of AKI after LT is multifactorial and includes recipient, graft, perioperative, and postoperative risk factors [4]. Pre-operative kidney dysfunction is often a reflection of the degree of liver dysfunction and an important risk factor for post-LT AKI [5, 6]. Perioperative intravascular depletion, severity of post-reperfusion syndrome, hemodynamic instability and nephrotoxic medications further increase the risk of AKI development [4, 7].

Despite improvements in organ preservation, surgery techniques, and immunosuppression protocols, the incidence of AKI after LT continues to be high, reaching 50% in some studies [8, 3]. In the first year after liver transplant, the development of AKI in the post-operative period is a major factor impacting organ survival [9, 10]. Several studies have shown reduced organ survival, especially when renal replacement therapy (RRT) is needed after transplant [11]. Development of chronic kidney disease (CKD) and/or accelerated progression to end-stage renal disease (ESRD) are also potential consequences of AKI after liver transplant [9, 12]. Progression to CKD occurs in 18.1% of patients after LT, and 4.8% of patients progress to ESRD in five years [13, 14].

Challenges in risk assessment and early management in patients developing AKI significantly contribute to worse prognoses in these patients. The limitations of serum creatinine (sCr) as a diagnostic marker for acute kidney injury are more evident in patients with liver disease due to malnutrition and decreased muscle mass [15–17]. Therefore, patients in the perioperative period of LT may benefit from more sensitive and specific biomarkers of kidney injury [18]. These biomarkers should be able to allow for early identification of AKI, ideally providing information about the etiology and helping to distinguish functional changes from structural damage.

Several biomarkers of early kidney injury have been identified and, although these novel AKI biomarkers have primarily been assessed in general intensive-care populations [19–21], they have also been applied in liver-transplant recipients [22–25]. NGAL is a small, secreted polypeptide that is upregulated in response to tubular injury and rapidly detectable in plasma and urine. Within 24 h after liver transplantation, plasma NGAL is a better predictor of AKI than serum creatinine [22–24, 26]. Additional studies with small numbers of patients have suggested that plasma NGAL can detect post-LT AKI as early as 1–2 h after reperfusion [22, 26]. Few studies have evaluated NGAL as a predictor of need for RRT and mortality in the perioperative period of LT.

In this study, we aimed to determine whether the pattern of NGAL urinary and plasma elevation in the perioperative period of LT could be a prognostic tool for determining AKI severity, progression, need for RRT and mortality.

## Methods

The University of Sao Paulo Ethics Committee approved the study under protocol numberCAAE:06636513.4.0000.0068. All clinical and research activities being reported are consistent with the Principles of the Declaration of Istanbul and with the Declaration of Helsinki. The protocol is registered among the clinical trials available at https://clinicaltrials.gov, with identifier NCT02095431. Patients were enrolled into the study after written informed consent was obtained from all participants as per the guidelines of the Institution’s Ethics Committee.

### Patients

**Fig. 1:**
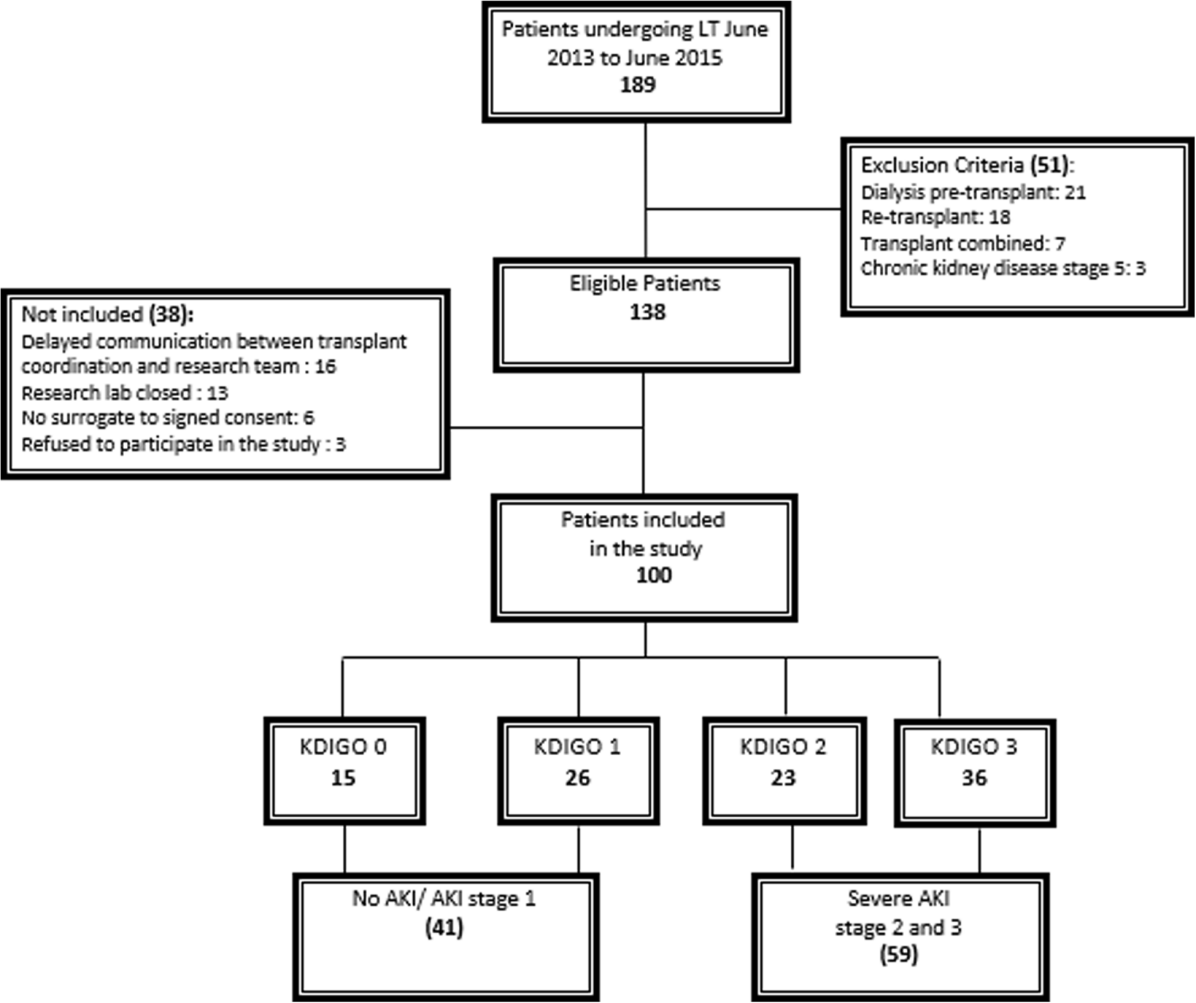
Flowchart of enrolled patients

During a 24-month period from June 2013 to June 2015, a total of 189 liver transplants were performed and 139 recipients were eligible for our study (Fig. 1). During the study period, all recipients older than 18 years old were screened. Patients were enrolled after written voluntary informed consent was obtained as per the guidelines of the Institution’s Ethics Committee. Exclusion criteria included pre-operative need for dialysis, combined transplant, chronic kidney disease stage 5, and previous kidney or liver transplant.

Patients were classified according to the etiology of end-stage liver disease (ESLD): hepatitis-B or -C cirrhosis, alcoholic cirrhosis, cryptogenic cirrhosis, acute hepatitis and miscellaneous causes. The miscellaneous group included patients with non-alcoholic steatohepatitis (NASH), autoimmune hepatitis, hemochromatosis, Budd–Chiari syndrome, biliary atresia, Wilson’s disease, primary sclerosing cholangitis, polycystic disease, biliary cirrhosis, and hepatocellular carcinoma.

The standard triple-drug immunosuppression regimen of tacrolimus with mycophenolate mofetil and steroids was used to prevent allograft rejection.

### Data collection

We recorded baseline kidney function and comorbid history from electronic medical records (EMRs). The following perioperative variables were included: main patient characteristics, intra-operative data, clinical follow-up in the first week after LT, need-for-RRT data, and outcomes.

The functional Model for End-stage Liver Disease (MELD) was calculated on the basis of serum bilirubin, international normalised ratio (INR) and serum creatinine, by the following formula:

$$ \mathrm{MELD}\kern0.5em \mathrm{score}\kern0.5em =\kern0.5em 0.957\times \mathrm{In}\left(\mathrm{creatinine}\kern0.5em \mathrm{mg}/\mathrm{dl}\right)+0.378\times \mathrm{In}\left(\mathrm{bilirubin}\kern0.5em \mathrm{mg}/\mathrm{dl}\right)+1.120\times \mathrm{In}\left(\mathrm{INR}\right)+0.643\times 10 $$

The liver transplant (LT) MELD was the sum of the functional MELD and the point score in special situations of transplant priority [27].

Blood and urine samples were collected simultaneously in the perioperative period of LT before induction of anesthesia, after portal reperfusion and at 6, 18, 24 and 48 h after surgery. We recorded vital signs, process of care, and lab results daily for 7 days after LT. Outcomes including maximum AKI severity stage, need for RRT and mortality were assessed in the intensive care unit (ICU), on hospital discharge and 60 days after enrolment.

### NGAL assessment

Blood samples were collected from central venous catheters or arterial lines, and urine through indwelling catheters. After collection, samples were immediately centrifuged – blood samples at 3000 rotations per minute (rpm) for 15 min and urine at 1000 rpm for 10 min – and stored at − 80 °C prior to analysis.

Quantitative NGAL levels were measured using particle-enhanced turbidimetric immunoassay (PETIA) for NGAL determination, according to the manufacturer’s instructions. The test uses an immunoturbidimetric method, in which polystyrene particles are coated with anti-NGAL antibody and, upon contact with the analyte in the sample, this results in turbidity proportional to the concentration of NGAL. Tests were performed in Labmax 560 equipment. Perioperative serum creatinine from the same samples as the NGAL measurement was analysed by chemiluminescence. All other serum creatinine values recorded from medical records were analysed by Jaffe method in the hospital’s central laboratory.

### Clinical outcomes

The primary outcome was AKI development during the first week of LT. Baseline renal function was defined as the lowest value in the prior three months and was used to assess renal recovery. AKI diagnosis was based on Kidney Disease: Improving Global Outcomes (KDIGO) [28]. For the reference serum creatinine value, we used the lowest value within 7 days before liver transplantation, and this value was used to determine AKI diagnosis. AKI stage was defined, according to KDIGO, as follows: stage 1 (1.5–1.9 times reference sCr or an increase of ≥0.3 mg/dl within 48 h), stage 2 (2.0–2.9 times reference sCr), stage 3 (3.0 times reference sCr or an increase of > 4.0 mg/dl). Patients with no AKI or stage 1 AKI were categorized as the no-AKI/mild-AKI group, whereas patients meeting the criteria for stage 2 or 3 AKI seven days after LT were identified as severe AKI. Secondary outcomes included need for RRT and mortality, assessed in the intensive care unit (ICU), on hospital discharge and at 60 days after enrolment.

### Statistical analysis

Continuous variables are presented as mean ± standard deviation (SD) or median (25th–75th percentiles) and were compared using one-way ANOVA or Kruskal–Wallis testing according to the Gaussian distribution. Categorical variables are presented as absolute numbers and percentages and were compared by the Chi-square test. *P* values were two tailed, and *P* < 0.05 was considered significant. Conventional receiver operating characteristic (ROC) curves were generated, and the area under the curve (AUC) was used to assess the ability of continuous variables to distinguish the categorical state: AKI development, need for dialysis and non-survival. The optimal cut-offs were determined by the best point of sensitivity versus specificity of the AUC (Youden index).

We compared time to AKI diagnosis based on biomarkers versus creatinine analyzed by chemiluminescence in the same sample collection. Time to diagnose based on sCr criterion was assessed by KDIGO stage 1 definition and biomarkers were determined by the cut-off value determined for plasma NGAL (PNGAL) and urine NGAL (UNGAL).

The perioperative covariates were tested in univariate analysis for their impact on AKI development, need for dialysis and non-survival. Factors associated with the outcomes at *P* < 0.01 were used to construct a multivariable model, in which the impact of each comorbidity or covariate was adjusted. In each model, UNGAL or PNGAL was included as a covariate. The SPSS (Statistical Package for Social Sciences) version 20 (Chicago, Illinois) software was used for the statistical analysis.

## Results

### Patient characteristics

Of 189 adult patients undergoing LT within the study period, 138 were eligible, and 100 were enrolled in the study. Figure 1 shows reasons for non-enrolment. Patients’ demographic and clinical characteristics are shown in Table 1. Baseline and reference serum creatinine values were similar in severe-AKI and no-AKI/mild-AKI groups. The estimated glomerular filtration rate (eGFR), based on the CKD Epidemiology Collaboration (EPI), corresponded to the baseline sCr, was similar within the groups. eGFR based on reference sCr was lower in the severe-AKI group.

**Table 1:** Clinical characteristics and outcomes in patients with and without moderate to severe AKI progression

PATIENT CHARACTERISTICS AND OUTCOMES	Total	No AKI/mild AKI	Severe AKI	*p*
N	100 (100%)	41 (41%)	59 (59%)	< 0.0001
Baseline Characteristics				
Age	58 (12)	57 (12)	53 (12)	0.01
Gender (Male)	64 (64%)	27 (42%)	37 (58%)	0.75
BMI	26 (4)	26 (4)	26.5 (5)	0.65
Non caucasian	14 (14%)	05 (36%)	09 (64%)	0.11
MELD functional	15 (11–19)	14 (10–17)	16 (12–22)	0.01
MELD LT	29 (29–29)	29 (29–29)	29 (29–32)	0.96
Liver Disease				
hepatitis C	46 (46%)	17(37%)	29(63%)	0.44
Alcoholic cirrhosis	13 (13%)	06(46%)	07(54%)	0.68
Cryptogenic cirrhosis	12 (12%)	05(42%)	07(58%)	0.96
acute hepatitis	06 (06%)	03(50%)	03(50%)	0.64
hepatitis B	04 (04%)	02(50%)	02(50%)	0.70
Other	19 (19%)	08(42%)	11(57%)	0.91
Comorbidities				
Hypertension	33(33%)	12(36%)	21(64%)	0.43
Diabetes mellitus	28 (28%)	14 (50%)	14 (50%)	0.26
Kidney function				
baseline sCr	0.77 (0.63–0.99)	0.77 (0.62–0.98)	0.77 (0.65–1.00)	0.87
reference sCr	0.78 (0.62–1.02)	0.77 (0.64–1.02)	0.80 (0.61–1.00)	0.96
Estimated GFR (CKD-EPI) by Scr ref.	78.65 (52–99)	93.50 (64–105)	69.40 (45–98)	0.013
Estimated GFR (CKD-EPI) by Scr base	99.65 (74–110)	99.25 (75–108)	108.52 (69–117)	0.67
Urine output first day after LT	475 (45)	608 (79)	383 (49)	0.01
Fluid balance first day after LT	+ 1535 (+ 500–2315)	+ 1010 (+ 367–1782)	+ 1655 (+ 890–2447)	0.008
Severity score indices				
SAPS	64 (60–72)	64 (11)	69 (15)	0.68
SOFA	13 (11–15)	12 (10–13)	14 (12–16)	0.001
process of care				
Anesthesia time	09:56 (01:59)	09:08 (01:33)	10:32 (02:04)	< 0.0001
HEPATECTOMY TIME (HH:MM)	03:11 (00:54)	02:54 (00:46)	03:27 (00:57)	0.003
warm ischemia time (HH:MM)	00:42 (1:11)	00:41 (1:21)	00:43 (1:52)	> 0.0001
cold ischemia time (HH:MM)	06:04 (01:55)	05:49 (02:06)	06:14 (01:46)	0.73
Red blood cELLS (unit)	2.39 (2.8)	1.45 (2.57)	3 (2.9)	0.002
outcomes				
Time with vasoactive drugs (days)	2 (1.78)	1(1.18)	2 (2)	0.01
Days of Mechanical ventilation	2 (1.82)	1 (0.57)	3 (2)	< 0.0001
Lenght of ICU stay (days)	9.81 (13)	5.59 (6.3)	12.75 (2)	0.003
Lenght of hospital stay (days)	29 (28)	19.17 (14.6)	36 (4.2)	< 0.0001
Need for Retransplant	11 (11%)	01 (09%)	10 (91%)	0.02
Need for RRT	36 (36%)	04 (10.5%)	34 (89.5%)	< 0.0001
60 day Mortality	21 (21%)	03 (14.3%)	18 (85.7%)	0.004

Data are expressed as n (%), mean (±), median and percentile (25–75) according to their distribution. Time of anesthesia expressed as hours: minutes. BMI (body mass index), MELD (model for end-stage liver disease), LT (liver transplant), ICU (intensive unit care), SAPS (Simplified Acute Physiology Score), SOFA (Sequential Organ Failure Assessment), RRT (renal replacement therapy), GFR (glomerular filtration rate), CKD- Epidemiology Collaboration

### AKI development

Based on the baseline ambulatory values, 34 patients were classified with acute kidney disease before surgery, of whom 23 (67.6%) were KDIGO stage 1. Based on the reference sCr, 85 patients developed AKI (stage 1/2/3). Patients developing no AKI or stage 1 AKI within the first 7 days of LT were summarized as the no-AKI/mild-AKI group 41 (41%), whereas patients meeting the criteria for stage 2 or 3 AKI group within the first 7 days of LT were summarized as the severe-AKI 59 (59%) (Fig. 1).

### Risk factors for AKI

Patients developing severe AKI were younger, had a higher functional MELD before surgery and higher Sequential-Organ--Failure (SOFA) scores on ICU admission (Table 1). Duration of anesthesia, hepatectomy and warm ischemia time were significantly prolonged in severe-AKI patients. In the first day after LT, urine output was significantly lower in the severe-AKI group and the cumulative fluid balance was higher, with a difference of approximately 600 ml between groups (*P* = 0.008).

### Performance of NGAL for diagnosis of AKI in the perioperative LT period

**Fig. 2:**
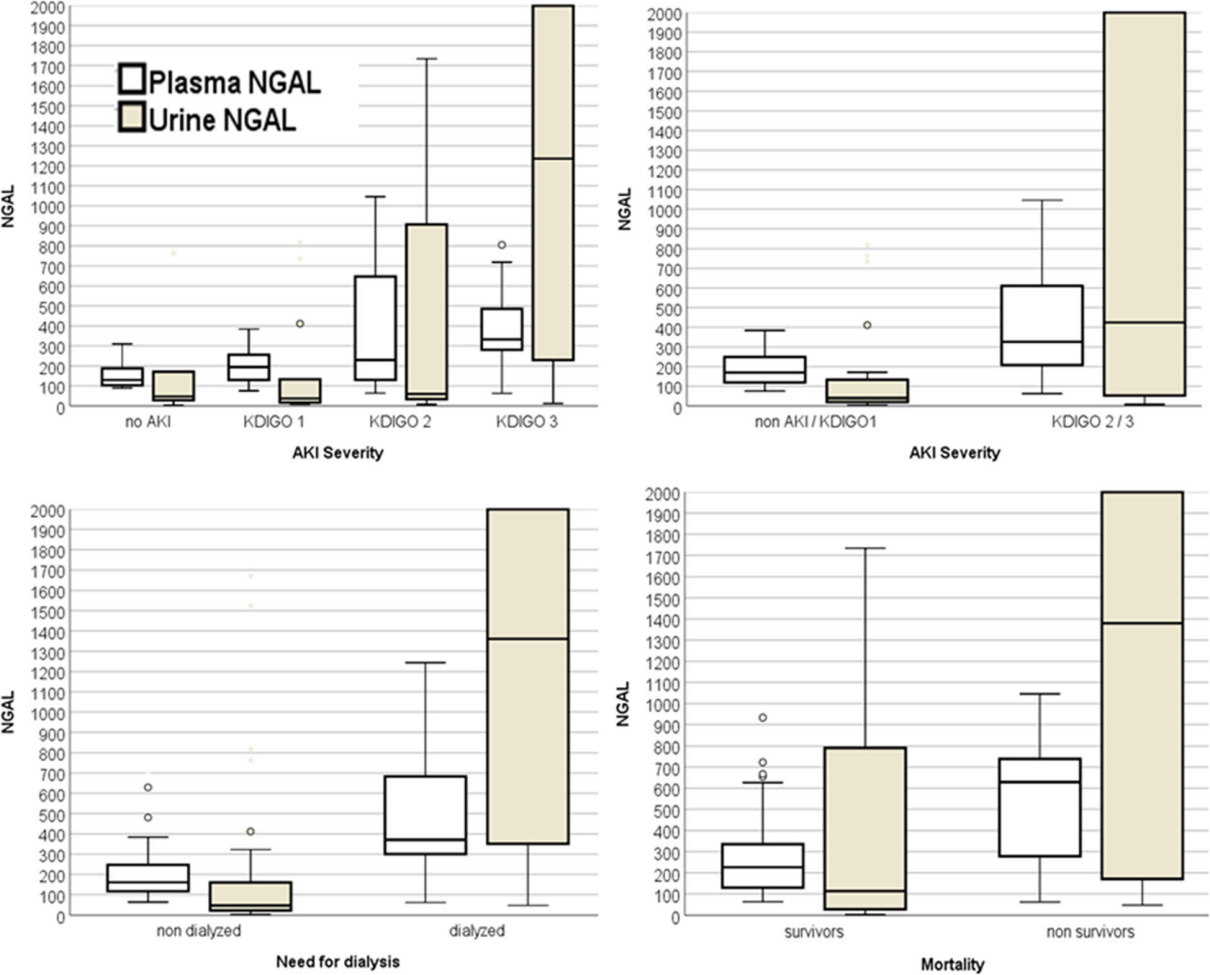
Median of the PNGAL eighteen hours after LT and UNGAL six hours after LT and outcome groups

Six hours after LT, severe-AKI patients had 10.6-fold higher UNGAL levels than those in the no-AKI/mild-AKI group, followed by a sustained significant difference for 24 h after surgery (Fig. 2). UNGAL achieved the best performance for predicting severe AKI at six hours after LT, with an AUC of 0.76 (95% CI 0.67–0.86). The best cut-off value was 136 ng/ml, with a sensitivity of 68%, specificity of 76%, and positive predictive value (PPV) of 80%.

Median PNGAL was higher in the severe-AKI versus no-AKI/mild-AKI group, reaching statistical significance six hours after LT; severe AKI 265 ng/ml vs. no-AKI/mild-AKI 138 ng/ml (*P* = 0.01). Eighteen hours after LT, PNGAL levels were approximately 2-fold higher in severe-AKI patients; median 326 ng/ml in AKI vs. 170 ng/ml in no-AKI /mild-AKI (Fig. 2). The best cut-off value for predicting AKI was 198 ng/ml, with a sensitivity of 87%, specificity 71% and PPV of 81% (Table 2).

**Table 2:** Progression of PNGAL and UNGAL in AKI, max KDIGO stage, need for RRT and mortality groups

PNGAL values						
	Pre-op. (100)	Intra-op. (95)	6 h (90)	18 h (87)	24 h (86)	48 h (74)
no AKI / mild AKI	172 (102–331)	196 (117–310)	138 (93–282)	170 (117–252)	156 (113–243)	116 (89–221)
severe AKI	185 (114–287)	232 (158–364)	265 (177–429)	326 (180–616)	356 (215–666)	369 (235–535)
P	0.82	0.50	0.047	0.004	< 0.0001	< 0.0001
no-RRT	166 (79–270)	192 (127–323)	183 (86–281)	168 (120–249)	173 (122–286)	134 (91–248)
need RRT	204 (115–465)	318 (204–407)	341 (242–510)	459 (306–749)	608 (333–749)	481 (292–689)
P	0.27	0.015	< 0.0001	< 0.0001	< 0.0001	< 0.0001
survivors	197.49 (117–326)	213.43 (129–337)	199.73 (110–343)	226.06 (129–335)	228.09 (134–368)	116.11 (117–326)
non-survivors	165 (98–249)	309 (105–389)	279.5 (183–529)	629.01 (256–759)	619.10 (301–689)	503.50 (281–697)
P	0.51	0.64	0.22	0.001	0.001	0.001
UNGAL values						
	Pre-op. (97)	Intra-op. (84)	6 h (87)	18 h (75)	24 h (71)	48 h (62)
no AKI / mild AKI	27 (17–244)	35 (16–402)	40 (18–143)	62 (32–380)	72 (26–248)	147 (44–338)
severe AKI	90 (23–254)	119 (26–1669)	424 (51–2802)	312 (55–1542)	329 (85–1290)	247 (50–1820)
P	0.90	0.008	< 0.0001	0.006	0.004	0.23
no-RRT	27(16–167)	26(15–98)	70(27–160)	78(35–262)	110(28–318)	70(18–317)
need RRT	179 (34–513)	602 (34–2912)	1361(603–2131)	855 (335–1979)	660 (195–3109)	667 (102–1650)
P	0.001	< 0.0001	< 0.0001	< 0.0001	0.001	0.002
survivors	33 (15–372)	47.10 (20–522)	113.10 (28–817)	131.20 (37–818)	147.80 (42–546)	214.80 (95–1608)
non-survivors	220.40 (49–549)	181.71 (42–3046)	1235.70 (77–3806)	309.69 (138–3718)	332.71 (109–3861)	3291 (340–3950)
P	0.02	0.11	0.005	0.06	0.09	0.0004

Data are expressed as median and percentile (interquartile range 25–75) as the nonparametric distribution UNGAL. The Shapiro–Wilk normality test was performed. AKI: acute kidney injury; RRT: renal replacement therapy

### Early diagnosis of AKI by biomarkers versus serum creatinine

**Fig. 3:**
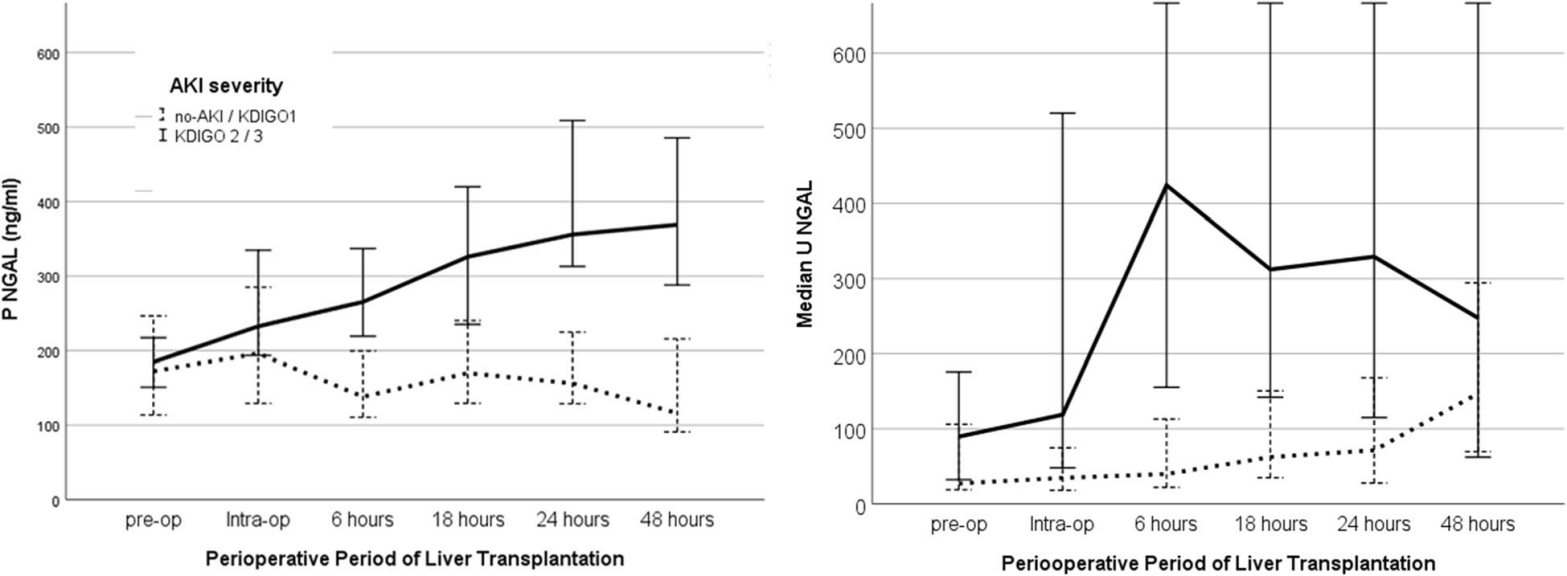
Median of the PNGAL and UNGAL in the perioperative liver transplant in no-AKI/ mild- AKI and severe-AKI groups

We evaluated time to AKI diagnosis based on NGAL cut-off values and sCr KDIGO criterion in the first 48 h after transplant. For this analysis, we used sCr measured by chemiluminescence in the same samples as the biomarkers. AKI diagnosis was reached in 72 patients by sCr, in 83 by PNGAL and in 65 by UNGAL levels. The median time based on PNGAL was 28 h earlier than sCr (Fig. 3), and UNGAL reached diagnosis 23 h earlier than sCr (Fig. 3).

### Performance of NGAL in determining the need for RRT in the perioperative LT period

Thirty-eight patients were dialysed during hospitalization, 34 in the first week after LT. The best performance of PNGAL in predicting the need for RRT was at 18 h, with an AUC of 0.84 (95% CI 0.74–0.93), sensitivity of 87% and a specificity of 83%. Using the cut-off value of 286 ng/ml, UNGAL was able to predict the need for RRT with the best performance at 6 h after LT, a cut-off of 210 ng/ml determining an AUC 0.85 (95% CI 0.77–0.93), with a sensitivity of 86% and the specificity of 80% (Table 2, Fig. 2).

Variables associated with the need for RRT in the univariate analysis were included in the multivariable logistic model: duration of anesthesia, SOFA score at ICU admission, urine output and fluid balance within 24 h of surgery (Table 3). In the model including UNGAL, the biomarkerwas the most powerful predictor for RRT need.

**Table 3:** Multivariable logistic models with PNGAL and UNGAL for AKI, need for RRT and mortality

VARIABLES	model pngal OR (IC 95%)	*P*	model ungal OR (IC 95%)	*p*
AKI				
Age (years)	0.9 (0.92–1.02)	0.25	0.98 (0.93–1.02)	0.4
duration anesthesia (hours)	1.54 (1.08–2.20)	0.16	1.61 (1.14–2.27)	0.007
Urine output first day after LT (ml)	0.99 (0.99–1.00)	0.28	26.5 (5)	0.65
Fluid balance first day after LT (ml)	1.00 (1.00–1.00)	0.07	0.99 (0.99–1.00)	0.12
sofa	1.12 (0.91–1.38)	0.27	16 (12–22)	0.01
ngal (ng/ml)	4.72 (0.87–25.39)	0.07	7.87 (1.2–39.70)	0.02
need rrt				
duration anesthesia (hours)	1.11 (0.79–1.56)	0.52	1.11 (0.75–1.69)	0.58
Urine output first day after LT (ml)	0.99 (0.99–0.99)	< 0.001	0.99 (0.99–0.99)	0.001
Fluid balance first day after LT (ml)	1.00 (1.00–1.00)	0.22	1.00 (1.00–1.00)	0.36
sofa	1.48 (1.13–1.94)	0.004	1.49 (1.10–2.02)	0.009
ngal (ng/ml)	2.74 (1.35–5.52)	0.005	35.28 (2.8–444)	0.006
MORTALITY				
duration anesthesia (hours)	1.48 (1.08–2.09)	0.01	21(64%)	0.43
MELD	1.00 (0.88–1.13)	0.95	14 (50%)	0.26
sofa	1.72 (1.23–2.42)	0.002	1.62 (1.16–2.26)	0.004
ngal (ng/ml)	2.20 (0.33–15)	0.41	6.10 (0.66–56)	0.11

AKI (acute kidney injury), OR (odds ratio), MELD (model for end-stage liver disease), SOFA (Sequential Organ Failure Assessment), RRT (renal replacement therapy)

### Performance of NGAL to predict mortality in the perioperative LT period

The 60-day mortality rate was 21% (21); 03 (14.3%) in no-AKI/mild-AKI group and 18 (85.7%) in the severe-AKI group (*P* = 0.005). The main causes of death were septic shock 07 (33.3%), primary graft dysfunction 03 (14.3%) and hemorrhagic shock 03 (14.3%). The best time point for using PNGAL levels to predict mortality was at 18 h after surgery, with an AUC of 0.76 (CI95% 0.60–0.92) for levels higher than 483 ng/ml. UNGAL was significantly higher in non-survivors even before surgery, in the pre-operative period. The median was 33 ng/dl in survivors versus 220 ng/dl in non-survivors (*P* = 0.002). Six hours after LT, a value higher than 242.06 ng/ml had an AUC of 0.81 (CI 0.65–0.97) for predicting mortality with sensitivity 86% and specificity 64% (Table 2 and Fig. 2). In the multivariable analysis, duration of anesthesia, SOFA on ICU admission and MELD were included in the model. The only independent predictor of mortality, in analyses including PNGAL or UNGAL, was the SOFA score (Table 3).

## Discussion

Acute kidney injury in the post-operative period of LT is associated with several short- and long-term complications. The short-term effects of AKI includs disturbances in acid-base and electrolyte homeostasis, azotemia, volume overload, increasingd duration of mechanical ventilation and ICU stay. The long-term effects are reflected in the higher rates of CKD development and decreased patient and graft survival.

The importance of early diagnosis of AKI is particularly significant in patients with chronic liver disease, for whom serum creatinine is known to be a worse marker of kidney function. Based on ambulatory values, 11% of our patients in the waiting list for liver transplant were CKD stage 3 or 4. It is likely that eGFR values in these patients are overestimating renal function, and an even higher proportion of patients had a severe degree of dysfunction. The complications of liver disease in these patients predispose them to oscillations of serum creatinine, and frequent episodes of AKI. Before surgery, 34 patients in our cohort already had an elevation in sCr higher than 0.3 mg/dl from the baseline ambulatory values and were considered as having pre-operative AKI. In these patients, NGAL levels were considerably higher than in patients without pre-operative AKI and continued to increase during intra-operative assessment. The high levels of NGAL at the initial pre-operative assessment were associated with the degree of injury in the post-operative period.

The potential of early biomarkers of kidney injury, such as NGAL, to detect AKI earlier has been shown in previous studies [23, 24, 29]. Nieman et al. [22], found that a PNGAL level higher than 139 ng/ml after portal reperfusion was a good predictor of AKI development; AUC of 0.79. In our study, UNGAL values after portal reperfusion and 6 h after surgery were good predictors of AKI within 7 days, with AUC of 0.67 and 0.76. However, NGAL is not specifically related to kidney injury; it is produced in many different tissues. Consequently, marked elevation can also occur in acute as well as chronic systemic inflammation. As such, the cut-off for determining kidney injury in this specific population with a higher degree of inflammation may differ to that applied in critically ill patients. In the meta-analysis by Haase et al. [30], which included 19 studies and 2538 patients, 487 (19.2%) developed AKI and NGAL was a good predictor of AKI with an odds ratio (OR) of 18.6 and an AUC of 0.81. Analyzing different populations, the best cut-off value found in those studies varied within the 100–270 ng/ml range, and the authors proposed a value of 150 ng/ml for diagnosing AKI [30]. In our cohort, despite the high severity of illness, our cut-off levels for AKI at 198 ng/ml for PNGAL and 136 ng/ml for UNGAL were within the range proposed in the meta-analysis.

We found UNGAL to be a better predictor of AKI development than PNGAL. Similar findings were reported by Baron-Stefaniak et al. [31]. Although not adjusting for other covariates, the authors found UNGAL to be a better predictor of AKI severity than PNGAL. In our study, UNGAL was the most powerful independent predictor of AKI, with an OR of 7.87.

A few studies [32] have compared the timing of AKI diagnosis by early biomarkers in the perioperative of LT. In a prospective study of 92 liver transplant recipients, Wagener et al. [24] demonstrated that the urinary-NGAL/urinary-creatinine ratio 3 h after liver transplant was a reliable marker of post-LT AKI and provided a diagnostic benefit of approximately two days compared to serum creatinine. We evaluated the timing of AKI diagnosis by the gold standard, sCr, and our cut-off values for UNGAL and PNGAL. Despite the high frequency of early AKI in our study, the presence of multiple risk factors in our population, including possibly ongoing kidney injury in the pre-operative period, NGAL was able to anticipate AKI detection and prediction of its severity by one day.

It is possible that NGAL levels, used in association with sCr, could contribute to the evaluation of the likelihood of early recovery, helping to distinguish functional from established structural damage. Our findings also showed UNGAL to be a good predictor of need for RRT and mortality. UNGAL was an independent predictor of need for dialysis, with a sensitivity of 86% and specificity of 80% (AUC 0.85). This finding suggests that early peak levels can be useful in deciding on early RRT initiation and influencing RRT method, e.g., continuous therapy in high-risk patients.

The high mortality rate in our cohort can be partially explained by the overall severity of patients, their older age and presence of comorbidities (hypertension, diabetes mellitus), higher MELD [22, 24, 33], and prolonged warm ischemia time. Several studies have shown an association between pre-LT MELD score and post-LT AKI [34–37]. Patients with high MELD scores are found to have a significantly increased risk of post-LT AKI and the development of CKD and ESDR [38]. Each minute of warm ischemia time is associated with an increase of 8–9% in the risk of non-recoverable renal function after LT [39, 40]. Bleeding and need for transfusion were frequent in our cohort and also associated with the increase in the mortality rate [41]. We found that UNGAL levels 6 h after liver transplant were a good predictor of mortality, with values higher than 483 ng/ml in 18 out of 21 of non-survivors (85%). Predicting patients with higher risk for mortality in early stages of liver transplant can help physicians to decide on appropriate management and possibly lead to improved outcomes.

Despite not being a multicentre study, this is the largest cohort for evaluating a biomarker perioperative of liver transplant. In this analysis, we used serum creatinine as a sole performance comparator for diagnosis of AKI, not applying urine output criteria. It is possible that some patients would have an earlier diagnosis based on urine volume and the delay in AKI diagnosis by KDIGO system decreased. Our incidence of AKI was high: 85% and 59 were AKI stage 2/3. Previous studies applying the RIFLE criteria have not considered 0,3 mg/dl serum creatinine criteria to define AKI [22, 24, 29, 32]. In our study, we applied the serum creatinine KDIGO definition as stated – 0.3 mg/dl within 48 h and 50% within 7 days – in contrast to most studies, which limited the time frame for diagnosis to 48 h [23, 31, 42]. Thus, in our analysis, similar to other analyses [8, 31], we have grouped no AKI with mild-AKI. Larger cohorts including less severely ill patients should be evaluated to confirm NGAL predictive ability to determine AKI severity, need for dialysis and mortality.

## Conclusions

NGAL is a promising biomarker for predicting AKI severity in patients undergoing liver transplant. The pattern of plasma- and urinary-NGAL elevation in the period perioperative of liver transplant allows earlier AKI diagnosis than KIDGO criteria based on serum creatinine. UNGAL was an independent predictor of AKI development and need for dialysis. Future studies should evaluate whether the clinical use of these biomarkers could improve patient outcomes.

## Data Availability

The anonymized dataset is held by the corresponding author and data may be made available in part for secondary analysis by third parties. Access will be granted upon reasonable request.

## Abbreviations

AKI: Acute kidney injury
AUC: Area under the curve
CKD: Chronic kidney disease
eGRF: Estimated glomerular filtration rate
EMR: Electronic medical records
EPI: Epidemiology Collaboration
ESLD: End-stage liver disease
ESRD: End-stage renal disease
ICU: Intensive care unit
INR: International normalised ratio
KDIGO: Kidney Disease: Improving Global Outcomes
LT: Liver transplant
MELD: Model for end-stage liver disease
NASH: Non-alcoholic steatohepatitis
NGAL: Neutrophil gelatinase-associated lipocalin
PNGAL: Plasma NGAL
PPV: Positive predictive value
ROC: Receiver operating characteristic
rpm: Rotation per minute
RRT: Replacement therapy
sCr: Serum creatinine
SD: Standard deviation
SOFA: Sequential Organ Failure
SPSS: Statistical Package for Social Sciences
UNGAL: Urinary NGAL
